# A Reinforced Whale Optimization Algorithm for Solving Mathematical Optimization Problems

**DOI:** 10.3390/biomimetics9090576

**Published:** 2024-09-22

**Authors:** Yunpeng Ma, Xiaolu Wang, Wanting Meng

**Affiliations:** 1 School of Information Engineering, Tianjin University of Commerce, Beichen, Tianjin 300134, China; 2 College of Science, Tianjin University of Commerce, Beichen, Tianjin 300134, China

**Keywords:** whale optimization algorithm, opposition-based learning strategy, CEC test functions, exploration and exploitation

## Abstract

The whale optimization algorithm has several advantages, such as simple operation, few control parameters, and a strong ability to jump out of the local optimum, and has been used to solve various practical optimization problems. In order to improve its convergence speed and solution quality, a reinforced whale optimization algorithm (RWOA) was designed. Firstly, an opposition-based learning strategy is used to generate other optima based on the best optimal solution found during the algorithm’s iteration, which can increase the diversity of the optimal solution and accelerate the convergence speed. Secondly, a dynamic adaptive coefficient is introduced in the two stages of prey and bubble net, which can balance exploration and exploitation. Finally, a kind of individual information-reinforced mechanism is utilized during the encircling prey stage to improve the solution quality. The performance of the RWOA is validated using 23 benchmark test functions, 29 CEC-2017 test functions, and 12 CEC-2022 test functions. Experiment results demonstrate that the RWOA exhibits better convergence accuracy and algorithm stability than the WOA on 20 benchmark test functions, 21 CEC-2017 test functions, and 8 CEC-2022 test functions, separately. Wilcoxon’s rank sum test shows that there are significant statistical differences between the RWOA and other algorithms

## 1. Introduction

An optimization problem entails finding the most favorable solution among various decision-making options while adhering to specific constraints. The problem is usually nonlinear and discrete, so an accurate model is difficult to establish, and so an optimization problem is usually called a “difficult” problem. As a branch of applied mathematics, optimization problems have found application in diverse fields such as military, engineering, and management. In this context, swarm intelligence optimization algorithms are employed to address such problems. Compared with traditional optimization algorithms, swarm intelligence algorithms do not require specific conditions for the objective function or constraints of the optimization problem. These algorithms can optimize without relying on exact formulas or mathematical models. Additionally, these algorithms exhibit adaptability to uncertain factors encountered during the optimization process. Extensive research has proven their effectiveness in solving optimization problems.

A swarm intelligence optimization algorithm is a global optimization technique that imitates the behavior of insects, animals, and birds in nature. With the continuous exploration of scholars, a series of swarm intelligence optimization algorithms have been proposed, and some representative partial algorithms are presented in Table 1.

This paper examines the whale optimization algorithm (WOA), a novel meta-heuristic algorithm inspired and proposed by the hunting behavior of humpback whales. The WOA has many advantages, including simple operation, fewer control parameters, and strong ability to escape the local optimum. These characteristics have motivated researchers to apply it to solve various practical problems, such as feature selection [11,12], workshop scheduling [13,14,15], and image segmentation [16,17,18]. However, when faced with high-dimensional and complex problems, the WOA exhibits certain limitations, such as slow optimization speed, low optimization accuracy, and inadequate search and development capabilities [19,20]. In order to enhance the performance of the WOA, many scholars have proposed improvements. Lee et al. [21] proposed a hybrid whale optimization algorithm based on the genetic algorithm and heat exchange optimization algorithm (GWOA-TEO), which use memory-based crossover operators and a memory-based position update mechanism for leading solutions to improve the search ability. In this study, the antagonistic learning strategy was used to generate the antithetical solution of the global optimal solution, which not only accelerates the speed of the algorithm but also improves the search ability of the algorithm. Prasad et al. [22] used logistic chaos mapping to adjust the control parameters of the WOA, and the problem of precocious convergence was effectively alleviated. In this study, the adaptive inertia coefficient was used to control the speed of the algorithm, which better balances the global search ability and local search ability of the algorithm. Sun et al. [23] developed a multi-population improved whale optimization algorithm (MIWOA), which leverages the current optimal individual and weighting center to refine the search process, boosting both search capability and convergence speed. The algorithm divides the population into superior and inferior groups, which maintains population diversity and enhances the exploration ability. In this study, the antagonistic learning strategy was used to generate the antithetical solution of the global optimal solution, which not only accelerates the speed of the algorithm but also enhances the search ability of the algorithm. By incorporating individual historical optimal information, the algorithm expands the potential search space, increases population diversity, and speeds up convergence to the global optimum. Li et al. [24] proposed a multi-objective whale optimization algorithm based on multi-leader guidance, which employs an opposition-based learning strategy to improve the distribution of the initial population. In this study, the oppositional learning strategy was used to generate the antagonistic solution of the global optimal solution, and it was observed that this strategy is more effective in enhancing the global optimum than in improving the initial population distribution. Islam et al. [25] combined the whale optimization algorithm with the multi-objective non-dominant sequencing technique to develop a hybrid algorithm called the Non-dominant Sequencing Whale Optimization Algorithm (NSWOA), which was applied to optimize the parameters of dynamic and static load controllers of islanded microgrids. Saha et al. [26] introduced the Cosine-Modified Whale Optimization Algorithm, which uses the cosine function to select the control parameter a and utilizes the correction factor to reduce the step size during the position update process, which appropriately balances the development and exploration ability of the algorithm. This study utilized an adaptive inertia coefficient to balance the algorithm’s exploration and exploitation abilities, and the individual historical optimal information and the global optimal solution are introduced, which makes the RWOA have stronger, balanced development and exploration capabilities. Liu et al. [27] employed an elite search strategy to enhance global optimization and introduced an adaptive variable speed strategy to balance the algorithm’s search and development capabilities. This study achieved a better balance between the development and search capabilities by incorporating individual historical optimal solutions, adaptive inertia coefficients, and enhanced global optimal solutions. Jin et al. [28] integrated the Eagle strategy [29] and uniform mutation into the whale optimization algorithm, which better balanced the global and local search capabilities. In [30], an adversarial learning strategy was introduced in the initial population generation to improve the diversity of algorithm. This studyusedan antagonistic learning strategy to refine the global optimal solution, and the experiments revealed that this approach is more effective in improving the global optimal solution. Furthermore, Lin et al. [31] proposed a niche hybrid heuristic whale optimization algorithm. Zong et al. [32] proposed a memory strategy based on fractional-order expansion to enhance the search ability of the whale optimization algorithm. Zhou et al. [33] developed a hybrid chameleon whale optimization algorithm (HWOA-CHM) inspired by the chameleon’s hunting mechanism, which improves the convergence accuracy of the algorithm. Shen et al. [34] divided the population into three sub-populations of equal size according to the fitness value of the individual, and each population was given a different update mechanism, which improved the global optimization ability of the algorithm. Although these variants of the whale optimization algorithm show improvements over the traditional WOA, it is important to note that no single optimization algorithm can perfectly solve all optimization problems, as stated by the no-free-lunch theorem [35]. Therefore, the pursuit of optimizing optimization algorithms remains a continuous and meaningful endeavor.

To address the limitations of the whale optimization algorithm, this paper proposes a reinforced whale optimization algorithm (RWOA), in which three corrections are made on the basis of the traditional WOA: Initially, an opposition-based learning strategy is incorporated to enhance the search for the global optimal solution. This strategy generates an opposing solution of the optimal solution, increasing the selectivity and aiding the algorithm find the target position faster. Secondly, an adaptive inertia coefficient is introduced in both the prey and bubble net stages. This dynamic control of the convergence speed enables individuals to focus more on utilizing their own position information in the early stages and approach the global optimal faster in the later stages. Additionally, the utilization of individual information is enhanced during the prey stage, which accelerates the algorithm’s convergence speed and improves the overall solution quality.

The main contributions of this paper are briefly summarized as follows:

(1) A new optimization algorithm called the RWOA is proposed. The algorithm incorporates an opposing learning strategy into the traditional WOA’s optimal solution, introduces adaptive inertia coefficients in the encircling prey and bubble net attack stages, and enhances the utilization of individual information during the encircling prey stage.

(2) The performance of the RWOA is verified using 23 standard test functions, 29 CEC-2017 test functions, and 12 CEC-2022 test functions.

(3) The effectiveness and superiority of the proposed algorithm is verified through the analysis of various statistical indicators, such as the mean and standard deviation. Additionally, the results of Wilcoxon’s rank sum test indicate a significant statistical difference between the RWOA and other comparison algorithms at a significance test level of 0.05.

The remainder of the paper is organized as follows: Section 2 reviews the basic whale optimization algorithms. The detailed procedure of the proposed RWOA is described in Section 3. Section 4 examines the performance of both the WOA and RWOA on various test functions, followed by a comparison and analysis of RWOA’s performance against other advanced optimization algorithms in Section 5. All experimental results are discussed in detail in Section 6. Finally, Section 7 summarizes this study’s findings and outlines future research directions.

## 2. Whale Optimization Algorithm (WOA)

In 2016, the whale optimization algorithm (WOA) was proposed by Mirjalili et al. [8], which was inspired by the predatory behavior of humpback whales. The algorithm can be divided into three main stages: encircling prey, bubble net attack, and searching for prey.

### 2.1. Exploitation Phase

During this stage of the WOA, the whales have found their target prey and approach the prey in a siege manner. The formulas in Table 2 are employed to simulate this predatory behavior.

### 2.2. Exploration Phase 

The WOA stipulates that when |A| ≥ 1, each whale will randomly select another whale from the population and learn from it, using Equations (1) and (2) to simulate this process.

(1) $$D_{\mathrm{rand}}^{t}=|C\cdot X_{\mathrm{rand}\;}^{t}-{\;X}_{i}^{t}|$$

(2) $$X_{i}^{t+1}{=X}_{\mathrm{rand}}^{t}-\;A\cdot D_{\mathrm{rand}}^{t}$$

Here, $X_{\mathrm{rand}}^{t}$ is the position vector of the randomly selected whale individual in the *t*th iteration, and $D_{\mathrm{rand}}^{t}$ is the distance between the *i*th whale individual in the *t*th iteration and the randomly selected whale individual.

## 3. A Reinforced Whale Optimization Algorithm

Although the WOA is simple to operate, has few parameters to be adjusted, has strong stability, and has a strong ability to escape the local optimum due to its unique update mechanism, it still has several drawbacks, such as low convergence accuracy and slow convergence speed. In order to overcome the shortcomings of the WOA, this paper proposes an enhanced version called the reinforced whale optimization algorithm (RWOA). The improvement strategy of the RWOA focuses on three main areas. Firstly, the opposition learning strategy is employed to generate antagonistic solutions for the global optimal solution after each iteration. Then, a greedy strategy selects the better fitness value as the global optimal solution. This approach expands the range of potential optimal solutions, which helps to accelerate the algorithm’s convergence speed. Secondly, based on the acceleration formula in the red-tailed hawk optimization algorithm, this paper improves the contraction bracketing mechanism and the spiral update position mechanism. In the early stage, whale individuals focus more on using their own information, and they get closer to their prey faster in the later stages. This approach better balances the algorithm’s exploration and exploitation capabilities. Thirdly, the original whale optimization algorithm lacks the full use of an individual’s valuable information. To address this limitation, this study added the utilization of an individual’s historical optimal information into the shrinking encircling mechanism of the WOA, so that the algorithm exhibits accelerated convergence speed and improved solution accuracy. The detailed improvement strategy is summarized as follows.

### 3.1. Opposition-Based Learning

Opposition-based learning (OBL) explores less examined areas of the solution space by creating solutions that are directly opposed to the current ones. These opposing solutions introduce new candidate solutions in diverse regions, thereby enhancing population diversity. These solutions offer alternative directions, which help address the limitations of the current solutions, broaden the search area, and enable the algorithm to swiftly converge to regions with high-quality solutions, thereby accelerating the process of finding the global optimum. OBL has been extensively utilized in various intelligent optimization algorithms [16,36,37,38,39,40,41] to enhance algorithm performance and has proven their effectiveness. Building on this idea, this study employed an opposition-based learning strategy to produce a new antagonistic solution for the global optimal solution after each iteration. This approach enlarges the algorithm’s search space and enhances solution diversity, aiding the algorithm in escaping local optima and thereby enabling it to identify the global optimal solution more quickly and accurately. The specific process is as follows:

(1) A complete update is performed on all individuals in the population, and each individual undergoes either a transformation to a better position or remains in the same position.

(2) The fitness value of each individual is calculated to find the global optimal solution, including the one before this iteration.

(3) The stochastic opposition-based learning strategy is employed to generate the antithetical solution (gbest′) of the global optimal solution (gbest), and the specific formula is as shown in Equation (3).

(3) gbest′=rand(1, dim) ×(lb+ub) − gbest

rand(1, dim) creates a matrix with 1 row and dim columns, where the elements are randomly generated from a uniform distribution in the range [0, 1]. Here, lb and ub represent the lower and upper limits of the feasible solution, respectively.

(4) Whether the generated antithetical solution (gbest′) falls within the specified upper and lower bounds is verified. If it exceeds these bounds, adjust it to the nearest limit value. The fitness value of gbest′ is calculated, and then the one with a favorable fitness value is selected as the global optimal solution (gbest) using a greedy strategy.

### 3.2. Adaptive Weight Strategy

**Figure 1:**
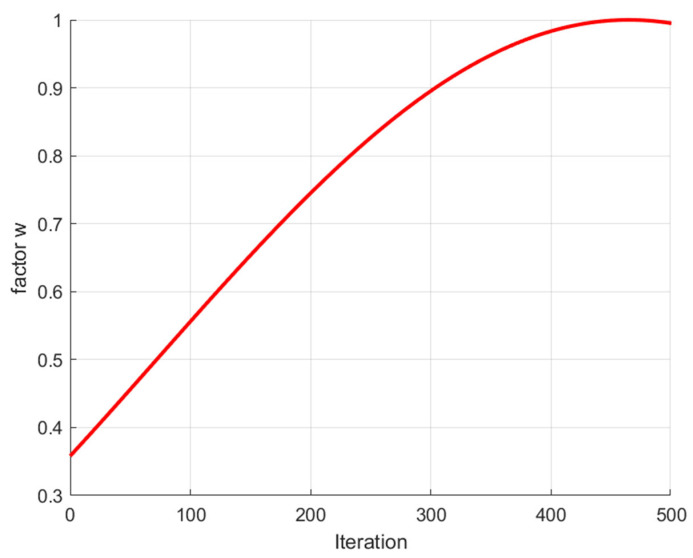
Trajectory diagram of w.

Many intelligent optimization algorithms have learned the idea of improving the inertia weight in the algorithm formula and achieving better results [40,42,43,44,45]; this study adopted the acceleration formula from the red-tailed hawk algorithm [46] to improve the two stages of the WOA’s encirclement of prey and bubble net attack. Introducing the dynamic adaptive inertia coefficient into the individual position update formulas allows individuals in the population to be less influenced by the global optimal solution in the early stages and to gradually increase this influence as iterations progress. Initially, individuals focus more on utilizing their own information, which reduces the tendency of the population to converge prematurely on inferior solutions and helps avoid local optima. In the later stages, the influence of the global optimal solution on the individual increases, which accelerates the convergence of the inferior solution to the optimal solution. This adjustment also effectively balances the algorithm’s global and local search capabilities. The expression for this improvement is as follows:

(4) $${w=\sin}^{2}(2.5\;-{\;t/T}_{\max})$$

where t represents the current iteration number, and $T_{\max}$ represents the maximum number of iterations of the algorithm. The trajectory of w over time is shown in Figure 1.

The improved formula is

(5) $$D_{i}^{t}=|C\cdot \mathrm{gbest}\;-{\;X}_{i}^{t}|$$

(6) $$D_{p}=|\mathrm{gbest}\;-{\;X}_{i}^{t}|$$

(7) $$X_{i}^{t+1}=\{\begin{array}{l} w\;\cdot \;\mathrm{gbest}\;-\;A\;\cdot {\;D}_{i}^{t},\;\;\;\;\;\;\;\;p\;<\;0.5\;(i)\;\;\;\\ w\;\cdot {\;\mathrm{gbest}+D}_{p}\;\cdot {\;e}^{\mathrm{bl}}\;\cdot \;\cos\left(2\pi l\right),\;p\;\geq \;0.5\;(\mathrm{ii})\;\; \end{array}$$

Here, w increases with the increase in the number of iterations t, which makes individuals pay more attention to self-exploration in the early stages of the algorithm and are more inclined to learn from society in the later stages of the algorithm. So, the algorithm approaches the global optimal solution more effectively with each iteration. The convergence speed of the algorithm is accelerated, and the global and local search capabilities of the algorithm are better balanced.

### 3.3. Improved Encircling Prey Mechanics

Inspired by [47,48], the comprehensive utilization of an individual’s information in the population, and the survival principle of “survival of the fittest” observed in the biological world, it is evident that individuals must continuously learn and accumulate knowledge to thrive. And individual learning can not only come from self-learning but also from learning from others. Self-learning is conducive to individual innovation, while learning from others accelerates individual knowledge accumulation. In this study, this learning mechanism was introduced into the prey encirclement stage of the whale optimization algorithm, which only introduces the use of the global optimal information in the population, while overlooking the potential of individual information. Through increasing the utilization of the historical optimal information of individuals in the population in the algorithm’s stage of surrounding prey, the individuals can not only learn from the optimal individuals of the population but also combine the use of their own optimal information in the past. By incorporating the utilization of individual information, our approach expands the potential search space within the population. This expansion leads to improved diversity among individuals, allowing for a more comprehensive exploration of the solution space. As a result, the algorithm can converge to the global optimal solution more quickly and with greater accuracy. The improved encircling prey mechanism is presented as follows:

(8) $$X_{i}^{t+1}=w\;\cdot \;\mathrm{gbest}\;-\;A\;\cdot {\;D}_{i}^{t}+A\;\cdot {\;|\mathrm{pbest}}_{i}^{t}-{\;X}_{i}^{t}|$$

Here, ${\mathrm{pbest}}_{i}^{t}$ is the historical optimal position of the ith individual after the t-th iteration.

This function is employed during the prey encirclement stage of the algorithm, which corresponds to the phase of identifying the global optimal solution. By incorporating individual information from within the population, this function effectively mitigates the risk of population convergence to local optimal solutions. Additionally, it enhances the diversity of the population, contributing to a more comprehensive exploration of the solution space.

**Figure 2:**
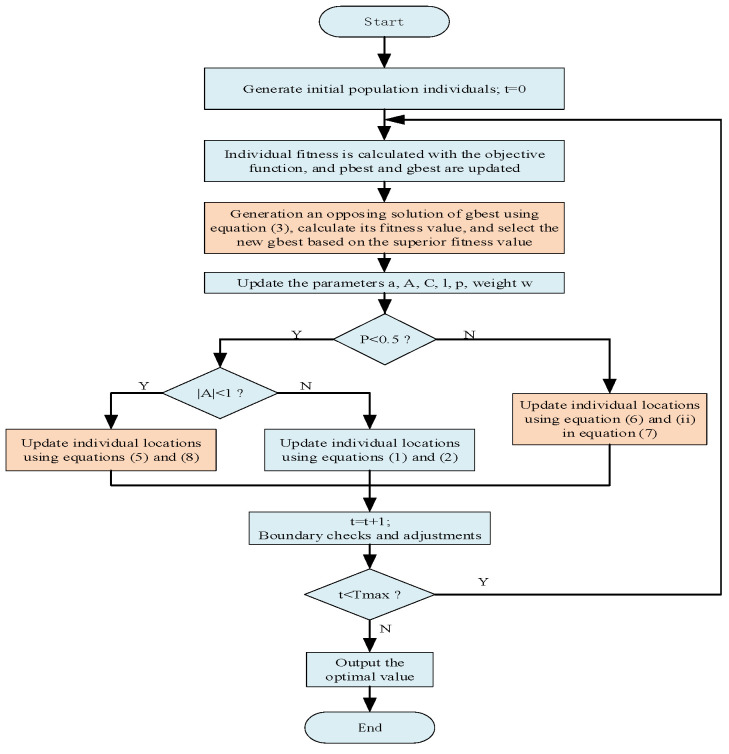
Flowchart of RWOA.

The pseudo-code for the RWOA is given in Algorithm 1, and an overall flowchart of the algorithm is given in Figure 2.
**Algorithm 1. Pseudo-code of RWOA**$1:\;\mathrm{Initialized}\;\mathrm{population}\;\mathrm{individuals}\;X_{i}$ (i = 1, 2, …, N) are randomly generated within the range of the problem space2: Each individual is assigned a value to the corresponding pbest. Calculate the fitness value of all individuals, find the best fitness value, and assign it to gbest.3: Initialize the parameters a, A, C, l, p, w4: t = 05: While t < T_max_ do6:    if *p* < 0.57:         if |A| < 18:           **Update each individual position using Equations** (**5**) **and** (**8**)9:         else if |A| ≥ 110:           Select a random search agent *X_rand_*11:           Update each individual position using Equations (1) and (2)12:         end if13:    else if *p* ≥ 0.514:         **Update each individual position using Equation** (**6**) **and** (**ii**) **in Equation** (**7**)15:    end if16:    **Update the individual historical optimal position pbest and its fitness value**17:    **Update the best optimal position gbest using the opposition-based learning strategy, Equation** (**3**)**; calculate the fitness value, and select the one with the best fitness value to reassign it to gbest**18:    Boundary checks and adjustments19:    t = t + 120:    Update the parameters a, A, C, l, p, w21: end while22: Output the global best solution (gbest)


### 3.4. Space Complexity Analysis

In this subsection, the space complexity of the algorithm is determined using Big O notation. The space complexity of the WOA is O(N × D), where N represents population size and D represents the dimension. According to the flowchart and pseudo-code of the RWOA, it can be seen that the complexity of the global optimal solution in the population after each iteration can be expressed as O(D). The adaptive inertia coefficient introduced in the RWOA does not increase the spatial complexity of the algorithm. And the computational complexity of the historical optimal solution of each individual can be expressed as O(N × D). The overall computational complexity of the RWOA is obtained by summing up these three complexities. Therefore, the final computational complexity of the RWOA is O(N × D). The results indicate that the space complexity of the RWOA is the same as that of the WOA, implying that the proposed algorithm does not increase the algorithm complexity.

## 4. Performance Testing of RWOA and WOA

In order to verify the effectiveness of the proposed RWOA, 23 benchmark functions were used to evaluate the performance of the RWOA in exploration, development, and minimization. Among the 23 benchmark functions, F1–F7 are uni-modal functions with only one extreme value used to evaluate the exploitation capability of the RWOA; F8–F13 are multi-modal functions with multiple local extreme values used to test the exploration performance of the RWOA; and F14–F23 are fixed-dimensional functions used to test the performance of the algorithm in low dimensions. In addition, 29 CEC-2017 test functions and 12 CEC-2022 test functions were used to further verify the performance of the RWOA.

All tests were performed on one machine: CPU: Intel(R) Core(TM) i7-8550U CPU @ 1.80 GHz 1.99 GHz, Windows11 operating system, and MATLAB R2021a. The population number was set as 40. The maximum number of iterations was set as 500. And the average value of the significance test level was 0.05. In order to reduce statistical errors, each algorithm was run independently 30 times.

### 4.1. Performance Testing on 23 Benchmark Functions

In this subsection, the performances of the RWOA and WOA are compared on 23 benchmark functions with 50 dimensions. The comparison is based on their exploitation ability, exploration ability, and algorithm convergence ability. For each test function, the RWOA and WOA were independently executed thirty times to find the global optimal solution. Then, the mean and standard deviation of the results from the thirty trials were calculated, with the mean denoted as Mean and the standard deviation denoted as SD. The mean represents the convergence accuracy of the algorithm, while the standard deviation represents its stability. It is important to note that smaller values for both the mean and standard deviation indicate better algorithm performance.

#### 4.1.1. Exploitation Capability Evaluation through Uni-Modal Functions

Uni-modal functions are those that have only one global optimal value in the interval, and these functions are suitable for evaluating the exploitation ability of optimization algorithms. Therefore, this study used the F1–F7 functions to assess the exploitation ability of the WOA and RWOA. The results of the experiment are recorded in Table 3, with the best performances highlighted in bold.

It can be clearly seen from Table 3 that for these functions, the proposed RWOA exhibits superior performance compared to the original WOA in terms of the maximum, mean, and standard deviation values. With the exception of the F7 function, the RWOA invariably achieves smaller minimum values, and the minimum value obtained by the two algorithms on the F7 function is almost the same. These experimental results clearly demonstrate that the RWOA has a stronger exploitation ability and better stability than the WOA.

#### 4.1.2. Exploration Ability Evaluation through Multi-Modal Functions

The effectiveness of both algorithm’s exploration ability was evaluated by testing them on multi-modal functions, which typically possess numerous local optimal values that increase exponentially with the problem size. In this study, the performances of the algorithms were also assessed using these multi-modal functions. Specifically, functions F8–F13 represent high-dimensional multi-modal functions, and their results are recorded in Table 4. Functions F14–F23 represent fixed-dimensional (low-dimensional) multi-modal functions, and their results are recorded in Table 5. 

As shown in Table 4, the proposed RWOA outperforms the WOA in all high-dimensional multi-modal functions. This suggests that the proposed algorithm exhibits enhanced exploration capabilities compared to the original algorithm. Furthermore, the values of all parameters are lower, indicating that the proposed algorithm is less prone to becoming trapped in local optima.

As can be seen from Table 5, the RWOA successfully identifies the global optimal value on F14–F20 for the fixed-dimension multi-modal function category, while also demonstrating greater stability than the WOA. The minimum values of the RWOA on the three test functions (F21–F23) are equal to those of the original WOA, and the mean values are similar as well. However, the maximum values and standard deviations are lower than the conventional WOA, indicating that the RWOA is generally better than the WOA. In summary, the RWOA has a better exploration capacity than the WOA on most test functions.

#### 4.1.3. Analysis of Statistical Test and Convergence Performance

Wilcoxon’s rank sum test was performed on the benchmark functions of the RWOA and WOA, and the results are presented in Table 2, Table 3 and Table 4. The obtained *p*-values of the test are below the significance test level, indicating a statistically significant difference between the two algorithms. Conversely, if the *p*-values were above the significance test level, it would suggest that there is no significant difference between the two algorithms. As indicated in Table 2, Table 3 and Table 4, at a significance test level of 0.05, except for functions F9, F18, and F20, the *p*-values for the remaining twenty functions are smaller than the significance test level. This suggests a significant difference between the RWOA and WOA under the statistical test. Combined with the previous analysis of the mean, standard deviation, and other indicators, it is clear that the proposed variant shows superior performance compared to the WOA on 23 benchmark functions.

**Figure 3:**
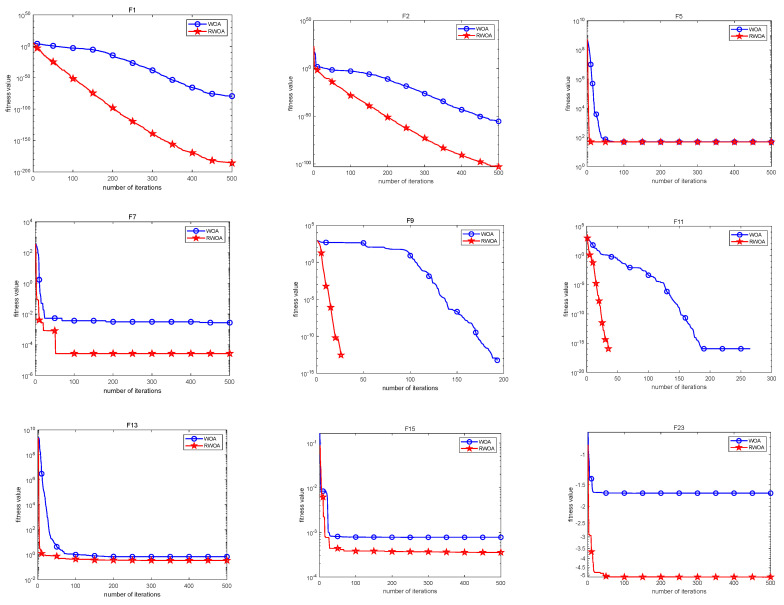
Comparison of convergence curves of WOA and RWOA.

In this section, simulation plots of some test functions are provided to compare the convergence accuracy and speed of the two algorithms. From these graphs, the convergence performances of the two algorithms can be compared more intuitively. The blue line is the convergence curve of the WOA, and the red line is the convergence curve of the RWOA. In examining Figure 3, it is evident that the RWOA exhibits superior convergence accuracy and speed compared to the WOA.

### 4.2. Performance Testing on CEC-2017

In this study, the performance of the proposed RWOA was tested on the CEC2017 benchmark functions with 10 dimensions. Among the 30 functions, the remaining 29 functions were selected for testing because F2 exhibits strong instability in high dimensions. CEC2017 benchmark functions are mainly divided into four categories: The uni-modal functions (F1–F3) have the characteristic of a narrow ridge, which is inseparable and smooth, and they are used to challenge the convergence ability of the algorithm. The simple multi-modal functions (F4–F10) offer numerous local optimal solutions to test the ability of the algorithm to jump out of the local optimum. The hybrid functions (F11–F20) exhibit minimal deviation between local optimal and global optimal values, evaluating the algorithm’s performance in navigating complex landscapes. And the synthesis functions (F21–F30) encompass all the characteristics of the previous categories, providing a comprehensive assessment of the algorithm’s overall performance. Compared to other test functions, the CEC2017 benchmark functions offer a broader range of functionality, better reflecting the algorithm’s performance.

A careful analysis of Table 6 reveals that the Mean value of the RWOA is less than that of the WOA on all 21 functions, and the SD value of the RWOA is less than that of the WOA on almost all functions. In addition, almost all functions showed significant statistical differences in the functions where the RWOA is better than the WOA, and the *p*-value of the statistical test is at the test level of 0.05. Based on these results, it is evident that the proposed variant exhibits superior performance over the WOA on CEC2017 test functions.

### 4.3. Performance Testing on CEC-2022

CEC-2022 encompasses a collection of 12 benchmark functions that serve as a standard for evaluating optimization problems. It is currently the most widely used and newest test set. This test set is mainly divided into four categories: uni-modal functions (F1), basic functions (F2–F5), mixed functions (F6–F8), and combined functions (F9–F12). These test functions are known for their inherent complexity, making them highly challenging for evaluating the performances of optimization algorithms. In this study, the performances of the original whale optimization algorithm and the improved whale optimization algorithm were compared on this test set, with the results for 10 dimensions presented here.

From Table 7, it can be found that on the 12 functions of CEC2022, there are 8 Mean values and SD values of the RWOA that are smaller than those of the WOA. Furthermore, on the functions that the RWOA is better than the WOA, the *p*-value of the statistical test shows a significant statistical difference at the test level of 0.05. Based on these findings, it is clear that the proposed variant showcases superior performance over the WOA on the CEC2022 test functions.

## 5. Comparison of Performance with Other Algorithms

### 5.1. Compare with TSA and ABC

In this study, the TSA and ABC were first chosen for comparison with the proposed RWOA on ten benchmark testing functions. The TSA and ABC have been verified in that they are effective optimization algorithms for solving mathematical problems in the literature [6]. Therefore, this paper refers to 10 testing function results from the literature [6], which are denoted in Table 8. For the fairness of the comparison, the experimental condition is the same as in the literature [6]. For the RWOA and WOA, the number of population individuals was set as 40, the maximum iteration was 500, and each algorithm was run independently 30 times. The detailed results are presented in Table 8.

The experimental results indicate that all algorithms can find the optimal value on function TF7. TSA obtains the lowest Mean and SD on functions TF1, TF2, and TF3, as well as the lowest Mean on TF9. ABC achieves the best performance on functions TF4 and TF6. Compared to the other algorithms, the RWOA displays the smallest Mean and SD on functions TF3, TF5, and TF8, along with the lowest SD on function TF9. Analysis indicates that the RWOA demonstrates strong stability and a notable convergence ability compared to other algorithms.

### 5.2. Comparison of the Performance of RWOA with Multiple Algorithms

To conduct a comprehensive assessment of the performance of the RWOA, 23 benchmark test functions were employed in 10, 50, and 100 dimensions. In addition, several state-of-the-art optimization algorithms were used as comparative algorithm, including the KH, DBO, CS, SCA, PSO, GWO, MPA, and WOA. To ensure fairness, the number of population individuals for each algorithm was set to 40. Additionally, the maximum number of iterations was fixed at 500. Each algorithm was run independently 30 times, and the different parameter values of each algorithm remained consistent with their respective original algorithm; details are shown in Table 9.

**Figure 4:**
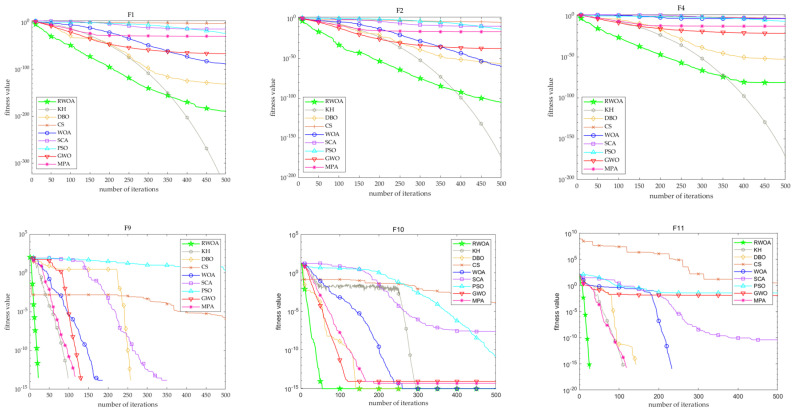
Convergence curves at dimension = 10.

As depicted in Table 10, the RWOA can still converge to a precise value in the low-dimensional case. With exceptions for functions F5 and F6, the RWOA consistently outperforms the WOA in terms of convergence accuracy, and the standard deviation of the RWOA is less than that of the WOA besides on function F6. The *p*-values of Wilcoxon’s rank sum test of the RWOA and WOA are almost less than 0.05 at the significance test level of 0.05, indicating that there is a significant statistically different between them, which proves the effectiveness of the proposed RWOA. The mean and standard deviation of the RWOA on functions F1-F4 are better than those of all algorithms except for KH, and there are significant statistical differences under the significance test level of 0.05. The RWOA stands out in terms of convergence accuracy and stability on functions F7, F8, F9, F10, and F11, surpassing other comparison algorithms, and the statistical test *p*-values below 0.05, indicating that the RWOA has a better performance than these algorithms. Additionally, it can be seen from Figure 4 that the RWOA achieves optimal solutions within its allowable range and exhibits a fast convergence speed, which indicates the stability and superiority of the improved algorithm in this paper.

As can be observed from Table 11, the mean values of the RWOA exhibit superior performance on functions F1–F4, second only to that of KH, indicating that the RWOA has a better performance in the single-peak function. The RWOA achieves the best mean values among the eight comparison algorithms on functions F7–F11, indicating its excellent performance in handling multi-modal functions. Additionally, although the accuracy of other functions may not be optimal, it is not significantly far from the optimal accuracy. At the same time, from the comparison of all standard deviations of each algorithm, the RWOA also has excellent performance in terms of standard deviation. Through the non-parametric statistical test, significant differences are observed between the RWOA and the eight algorithms across nearly all functions at a significance level of 0.05. Overall, the RWOA has better stability and convergence accuracy than other algorithms.

**Figure 5:**
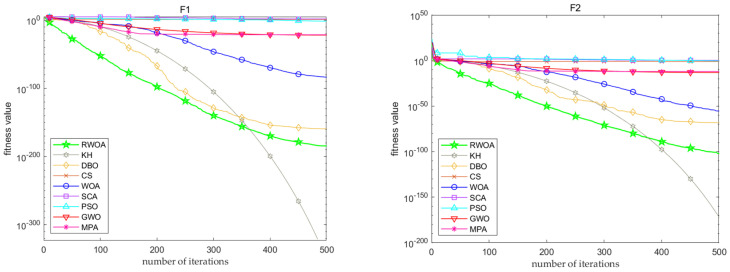

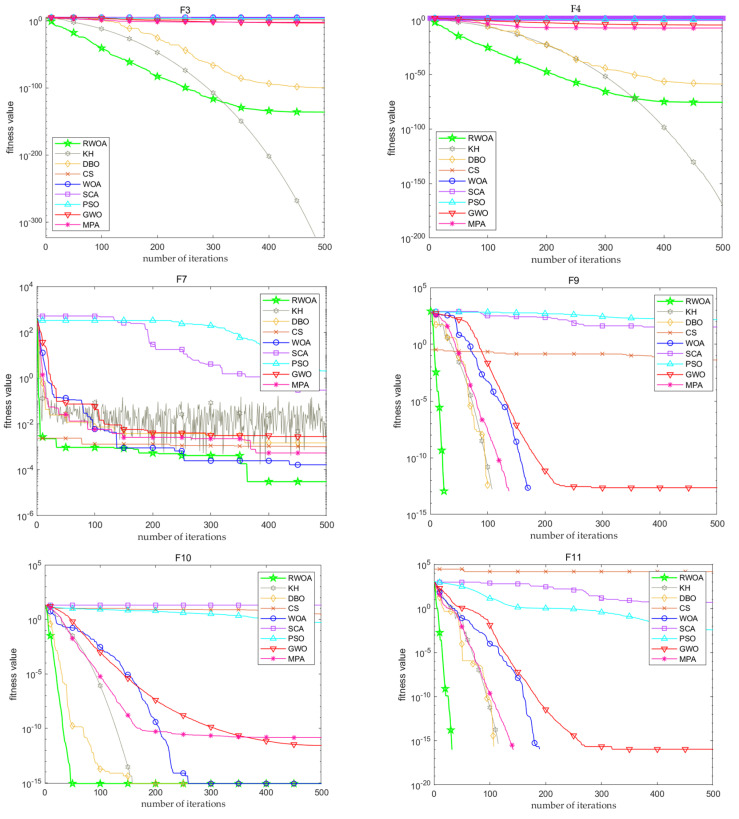
Convergence curves at dimension = 50.

Furthermore, in order to better illustrate the convergence performance of various algorithms and compare the convergence speed of the algorithms more intuitively, the convergence speed curves of each algorithm were simulated in this study, as shown in Figure 5. From the figures, the convergence speed and convergence accuracy of the proposed algorithm exhibit a better performance than those of the other algorithms on most functions.

After careful analysis of Table 12, it can be concluded that the mean obtained by the RWOA on functions F1–F4 is second only to that of KH, while the Mean value of the RWOA is the best on functions F6–F13. This indicates that the RWOA can find the global optimal solution more effectively than other algorithms when dealing with high-dimensional problems. The RWOA consistently exhibits the smallest standard deviation (SD) across nearly all functions, which proves that the RWOA has superior stability compared to the other algorithms in handling high-dimensional problems. The *p*-values obtained by the non-parametric statistical test show that there are statistical differences between the eight algorithms and the RWOA in almost all functions when the significance test level is 0.05.

**Figure 6:**
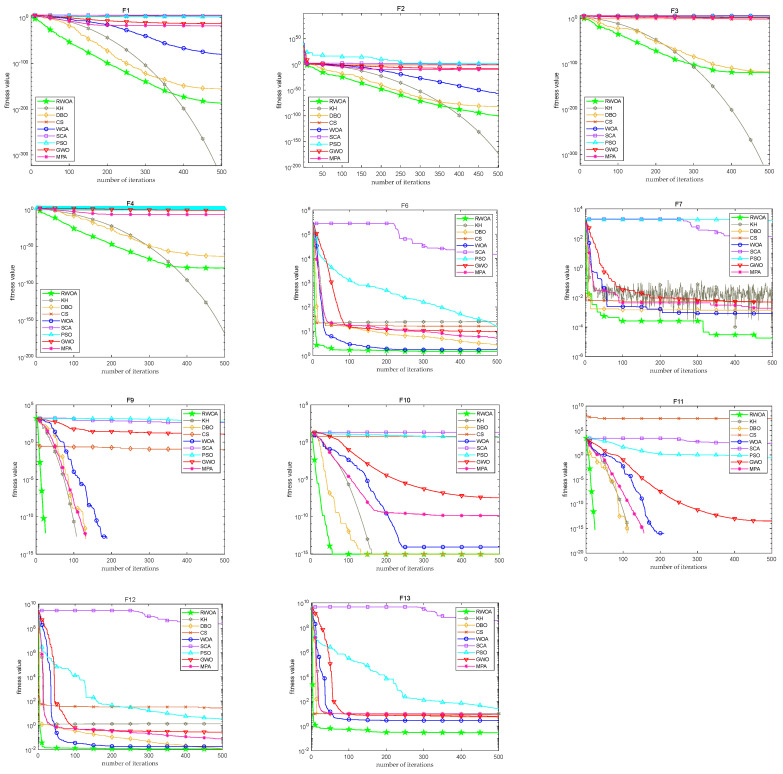
Convergence curves at dimension = 100.

The simulation curves of the algorithm’s convergence speed in 100 dimensions are depicted in Figure 6. These graphs clearly demonstrate that the RWOA has obvious advantages, as it exhibits a faster convergence speed and higher convergence accuracy compared to those of the other algorithms.

## 6. Discussions

In this paper, a new algorithm called the reinforced whale optimization algorithm (RWOA) is proposed. This algorithm incorporates adaptive coefficients and opposition-based learning strategies, and additionally, population information is integrated into the encircling prey update formula. To evaluate the performance of the RWOA, extensive experiments were conducted on 23 benchmark functions, 29 CEC-2017 test functions, and 12 CEC-2022 test functions, and the following conclusions are obtained:

(1) Among the 23 benchmark functions, the RWOA has smaller Mean and SD values compared to the WOA for all uni-modal test functions except function 7. The experimental results indicate that the RWOA exhibits a stronger development ability and better stability than the WOA. On all multi-modal test functions, the RWOA also demonstrates smaller Mean and SD values than the WOA, highlighting its enhanced exploration ability and reduced likelihood of local optima. On the fixed-dimension functions, the RWOA can find the global optimal value and has better stability than the WOA. The results of Wilcoxon’s rank sum test reveal that more than 85% of the *p*-values for the 23 benchmark functions are below the threshold at a significance level of 0.05. This indicates a significant difference between the RWOA and WOA.

(2) Among the 29 CEC2017 benchmark functions, the Mean value of the RWOA was found to be lower than that of the WOA for 21 functions, and the SD value of the RWOA is lower than that of the WOA for nearly all functions. In addition, the *p*-values of the statistical test show significant difference across almost all functions, at a significance level of 0.05.

(3) Among the 12 CEC2022 benchmark functions, the RWOA has smaller Mean and SD values than the WOA for 8 functions. Furthermore, on the function that the RWOA is better than the WOA, the *p*-values of the statistical test show a significant statistical difference at the test level of 0.05.

(4) The RWOA was compared to eight state-of-the-art optimization algorithms on 23 benchmark functions at dimensions of 10, 50, and 100, obtaining the following results:

(4.1) Dimensions 10 and 50: The Mean and SD values of the RWOA are smaller than those of all algorithms except KH for functions F1–F4. The RWOA achieves the best mean values among the eight comparison algorithms for functions F7–F11. Although the accuracy for other functions may not be optimal, it is not significantly lower than the best. The RWOA also performs exceptionally well in terms of the standard deviation. Non-parametric statistical tests revealed significant differences between the RWOA and the eight algorithms for nearly all functions at a significance level of 0.05. According to the convergence curve, the RWOA outperforms the other eight algorithms, demonstrating superior convergence speed and accuracy.

(4.2) Dimension 100: The RWOA achieves the lowest mean on functions F1–F4, second only to that of KH, and provides the best mean values for functions F6–F13. It consistently shows the smallest standard deviation across nearly all functions. Non-parametric statistical tests revealed significant differences between the RWOA and the eight algorithms for almost all functions at a 0.05 significance level. The convergence curve indicates that the RWOA has superior convergence speed and accuracy compared to the other algorithms.

## 7. Conclusions

To address the shortcomings of the whale optimization algorithm, such as slow convergence speed and low accuracy, a reinforced whale optimization algorithm is proposed. This new algorithm introduces adaptive coefficients and adversarial learning strategies, which improve the convergence speed and better balance the algorithm’s exploration and exploitation capabilities. Additionally, the utilization of population information in the contraction update formula is increased, accelerating the algorithm’s convergence speed and enhancing solution quality. To evaluate the performance of the RWOA, it was tested on 23 benchmark functions, 29 CEC-2017 test functions, and 12 CEC-2022 test functions. Statistical test indicators like the Mean and SD show that the RWOA demonstrates a higher convergence accuracy and greater stability compared to eight comparison algorithms across most test functions. The convergence speed simulation curves of the RWOA compared with the eight algorithms reveal that the RWOA converges faster and with higher accuracy. The non-parametric Wilcoxon sign rank sum test indicates a significant statistical difference between the RWOA and the other eight algorithms at the 0.05 significance level. Overall, the RWOA balances the exploration and development capabilities of the algorithm without increasing its complexity, achieving a faster convergence speed and higher accuracy.

In the future, based on the RWOA, a new multi-objective RWOA will be designed to solve multi-objective optimization problems and applied to address practical optimization problems.

## Figures and Tables

**Table 1:** Introduction to algorithms.

Algorithm	Pros and Cons	Year	Refs.
PSO	It has a strong global search ability and fast convergence speed, but it easily falls into the local optimal solution.	1995	[1]
ABC	A good balance of exploration and exploitation capabilities. However, it converges slowly and is sensitive to parameter configurations.	2005	[2]
CS	The model is simple, has few parameters, and is highly versatile, but it easily falls into local optimum.	2009	[3]
KHA	Strong global search ability, fast convergence speed, and good robustness.	2012	[4]
GWO	Its parameters are few, and the convergence speed is fast, but the algorithm easily matures early, and the convergence accuracy is low.	2014	[5]
TSA	It is easy to operate and provides a good balance between exploration and development capabilities. However, it is very sensitive to parameter selection and incurs high computational costs for high-dimensional problems.	2015	[6]
SCA	The structure is simple, and the calculation efficiency is high, but the convergence accuracy is low.	2016	[7]
WOA	The operation is simple, the control parameters are few, and the ability to jump out of the local optimum is strong, but the optimization speed is slow, the accuracy is low, and the exploration and development ability of the algorithm is poor.	2016	[8]
MPA	It has few parameters, has a simple structure, and is easy to implement with high calculation accuracy, but it easily falls into the local optimum and has a poor balance of mining and exploration, poor convergence speed, and solution quality.	2020	[9]
DBO	It has strong stability, fast search speed, and high accuracy, but it easily falls into the local optimum.	2022	[10]

**Table 2:** Location updates in the development phase.

Tactics	Update Formulas	Condition
encircling prey	$D_{i}^{t}=|C\;\cdot \;\mathrm{gbest}\;-X_{i}^{t}|$	*p* < 0.5, |A| < 1
	$X_{i\;}^{t+1}=\mathrm{gbest}\;-\;A\;\cdot {\;D}_{i}^{t}$	
	${A=2\alpha r}_{1\;}-\;\alpha$	
	$\alpha =2\;-{\;2t/T}_{\max}$	
	${C=2r}_{2}$	
bubble net attack	$X_{i}^{t+1}{=\mathrm{gbest}+D}_{p}\;\cdot {\;e}^{\mathrm{bl}}\;\cdot \;\cos(2\pi l)$	*p* ≥ 0.5
	$D_{p}=|\mathrm{gbest}\;-{\;X}_{i}^{t}|$	

Note: $X_{i}^{t}$ represents the position vector of the *i*th individual in the *t*th iteration (here, i = 1,2, …, N; N is the total number of whales); gbest represents the position vector of the target prey, A and C are coefficients; $r_{1}$, $r_{2}$, l, and p are random numbers between [0, 1]; α is decreasing linearly from 2 to 0; $D_{p}$ indicates the distance of the *i*th whale to the prey; b is a constant; t represents the current iteration number; and Tmax represents the maximum number of iterations for the population.

**Table 3:** Experimental results of RWOA and WOA on seven uni-modal functions.

Functions	RWOA				WOA				
	Max	Mean	Min	SD	Max	Mean	Min	SD	*p*-Values
F1	**2.36 × 10^−180^**	**1.12 × 10^−181^**	**4.68 × 10^−196^**	**0.00**	4.38 × 10^−80^	1.52 × 10^−81^	4.63 × 10^−90^	7.99 × 10^−81^	**1.73 × 10^−6^**
F2	**7.59 × 10^−101^**	**3.10 × 10^−102^**	**6.03 × 10^−107^**	**1.40 × 10^−101^**	9.61 × 10^−52^	5.07 × 10^−53^	3.71 × 10^−59^	1.84 × 10^−52^	**1.73 × 10^−6^**
F3	**1.25 × 10^−121^**	**4.43 × 10^−123^**	**4.01 × 10^−143^**	**2.29 × 10^−122^**	2.38 × 10^5^	1.74 × 10^5^	1.10 × 10^5^	3.58 × 10^4^	**1.73 × 10^−6^**
F4	**2.95 × 10^−72^**	**9.90 × 10^−74^**	**1.24 × 10^−83^**	**5.39 × 10^−73^**	92.30	61.30	0.28	30.8	**1.73 × 10^−6^**
F5	**48.20**	**47.60**	**46.90**	**0.31**	48.60	47.90	47.00	0.43	**5.32 × 10^−3^**
F6	**0.81**	**0.45**	**6.76 × 10^−2^**	**0.18**	1.62	0.72	0.14	0.29	**1.89 × 10^−4^**
F7	**1.81 × 10^−4^**	**4.36 × 10^−5^**	9.05 **× 10**^−6^	**4.17 × 10^−5^**	1.82 × 10^−2^	4.38 × 10^−3^	**7.87** × **10^−6^**	4.90 × 10^−3^	**1.92 × 10^−6^**

**Table 4:** Experimental results of RWOA and WOA on six high-dimensional multi-modal functions.

Function	RWOA				WOA				
	Max	Mean	Min	SD	Max	Mean	Min	SD	*p*-Values
F8	**−2.00 × 10^4^**	**−2.09 × 10^4^**	**−2.09 × 10^4^**	**2.26 × 10^2^**	−1.26 × 10^4^	−1.72 × 10^4^	−2.09 × 10^4^	2.80 × 10^3^	**1.73 × 10^−6^**
F9	0.00	0.00	0.00	0.00	0.00	0.00	0.00	0.00	1.00
F10	**8.88 × 10^−16^**	**8.88 × 10^−16^**	8.88 × 10^−16^	**0.00**	7.99 × 10^−15^	4.91 × 10^−15^	8.88 × 10^−16^	2.23 × 10^−15^	**3.17 × 10^−6^**
F11	**0.00**	**0.00**	**0.00**	**0.00**	0.21	2.15 × 10^−2^	0.00	5.74 × 10^−2^	**0.13**
F12	**2.40 × 10^−2^**	**1.27 × 10^−2^**	**1.49 × 10^−4^**	**5.57 × 10^−3^**	3.51 × 10^−2^	1.51 × 10^−2^	6.04 × 10^−3^	7.27 × 10^−3^	**0.22**
F13	**0.62**	**0.31**	**6.74 × 10^−2^**	**0.14**	1.76	0.78	0.28	0.32	**2.35 × 10^−6^**

**Table 5:** Experimental results of RWOA and WOA on ten fixed-dimensional multi-modal functions.

Function	RWOA				WOA				
		RWOA				WOA			
	Max	Mean	Min	SD	Max	Mean	Min	SD	*p*-Values
F14	10.80	1.82	1.00	1.87	10.80	2.96	1.00	3.25	**0.15**
F15	6.35 × 10^−4^	3.32 × 10^−4^	3.08 × 10^−4^	6.45 × 10^−5^	7.64 × 10^−3^	8.64 × 10^−4^	3.10 × 10^−4^	1.34 × 10^−3^	**4.73 × 10^−6^**
F16	−1.03	−1.03	−1.03	4.35 × 10^−6^	−1.03	−1.03	−1.03	8.74 × 10^−10^	**1.73 × 10^−6^**
F17	0.40	0.40	0.40	2.15 × 10^−5^	0.40	0.40	0.40	1.32 × 10^−5^	**1.75 × 10^−2^**
F18	3.00	3.00	3.00	1.16 × 10^−4^	3.00	3.00	3.00	5.42 × 10^−5^	0.67
F19	−3.85	−3.86	−3.86	2.69 × 10^−3^	−3.850	−3.86	−3.86	4.69 × 10^−3^	**0.13**
F20	−3.02	−3.21	−3.32	9.51 × 10^−2^	−3.05	−3.21	−3.32	9.57 × 10^−2^	0.88
F21	**−5.05**	−7.11	−10.2	**2.45**	−2.63	−8.46	−10.20	2.68	**1.11 × 10^−2^**
F22	**−5.08**	−6.97	−10.4	**2.52**	−2.77	−7.79	−10.40	3.08	**0.26**
F23	**−5.12**	−7.25	−10.5	**2.57**	−1.68	−7.83	−10.50	3.25	**5.45 × 10^−2^**

**Table 6:** Experimental results of RWOA and WOA on CEC-2017.

Function	RWOA				WOA				
	Max	Mean	Min	SD	Max	Mean	Min	SD	*p*-Values
CECF1	6.02 × 10^9^	2.08 × 10^9^	4.24 × 10^8^	1.07 × 10^9^	4.99 × 10^8^	4.15 × 10^7^	3.81 × 10^6^	9.18 × 10^7^	1.73 × 10^−6^
CECF3	6.91 × 10^3^	**3.06 × 10^3^**	1.57 × 10^3^	**1.22 × 10^3^**	3.71 × 10^4^	6.54 × 10^3^	7.37 × 10^2^	7.83 × 10^3^	**9.78 × 10^−2^**
CECF4	6.51 × 10^2^	4.99 × 10^2^	4.16 × 10^2^	**58.00**	6.15 × 10^2^	4.49 × 10^2^	4.01 × 10^2^	59.70	2.26 × 10^3^
CECF5	5.92 × 10^2^	5.70 × 10^2^	5.48 × 10^2^	**11.90**	6.23 × 10^2^	5.60 × 10^2^	5.18 × 10^2^	22.70	5.98 × 10^−2^
CECF6	6.62 × 10^2^	**6.37 × 10^2^**	6.08 × 10^2^	**10.90**	6.95 × 10^2^	6.42 × 10^2^	6.17 × 10^2^	16.90	**0.13**
CECF7	8.30 × 10^2^	7.96 × 10^2^	7.66 × 10^2^	**14.40**	8.72 × 10^2^	7.85 × 10^2^	7.46 × 10^2^	30.00	5.98 × 10^−2^
CECF8	8.62 × 10^2^	**8.37 × 10^2^**	8.26 × 10^2^	7.71	8.76 × 10^2^	8.46 × 10^2^	8.19 × 10^2^	16.90	**1.75 × 10^−2^**
CECF9	1.52 × 10^3^	**1.29 × 10^3^**	1.03 × 10^3^	**1.30 × 10^2^**	2.94 × 10^3^	1.64 × 10^3^	1.09 × 10^3^	5.01 × 10^2^	**5.71 × 10^−4^**
CECF10	2.82 × 10^3^	**2.17 × 10^3^**	1.42 × 10^3^	**3.41 × 10^2^**	2.91 × 10^3^	2.21 × 10^3^	1.51 × 10^3^	3.72 × 10^2^	0.59
CECF11	1.31 × 10^3^	**1.22 × 10^3^**	1.14 × 10^3^	**43.70**	1.57 × 10^3^	1.26 × 10^3^	1.13 × 10^3^	1.12 × 10^2^	**0.12**
CECF12	1.62 × 10^7^	**6.33 × 10^6^**	1.44 × 10^5^	**3.94 × 10^6^**	2.23 × 10^7^	6.63 × 10^6^	9.59 × 10^4^	7.00 × 10^6^	0.89
CECF13	3.59 × 10^4^	**1.15 × 10^4^**	3.04 × 10^3^	**7.62 × 10^3^**	3.46 × 10^4^	1.33 × 10^4^	2.07 × 10^3^	1.11 × 10^4^	**0.37**
CECF14	5.17 × 10^3^	**2.22 × 10^3^**	1.50 × 10^3^	**9.61 × 10^2^**	5.95 × 10^3^	2.85 × 10^3^	1.49 × 10^3^	1.67 × 10^3^	**0.36**
CECF15	1.93 × 10^4^	**6.15 × 10^3^**	1.65 × 10^3^	**4.26 × 10^3^**	4.85 × 10^4^	1.26 × 10^4^	2.59 × 10^3^	9.97 × 10^3^	**1.04 × 10^−3^**
CECF16	2.24 × 10^3^	**1.98 × 10^3^**	1.73 × 10^3^	**99.60**	2.17 × 10^3^	1.90 × 10^3^	1.64 × 10^3^	1.49 × 10^2^	**2.30 × 10^−2^**
CECF17	1.83 × 10^3^	**1.80 × 10^3^**	1.76 × 10^3^	**15.80**	1.94 × 10^3^	1.83 × 10^3^	1.75 × 10^3^	57.20	**6.04 × 10^−3^**
CECF18	3.72 × 10^4^	**1.35 × 10^4^**	3.30 × 10^3^	**9.31 × 10^3^**	4.25 × 10^4^	2.04 × 10^4^	3.30 × 10^3^	1.19 × 10^4^	**2.18 × 10^−2^**
CECF19	6.24 × 10^5^	1.03 × 10^5^	6.38 × 10^3^	**1.44 × 10^5^**	1.37 × 10^6^	9.76 × 10^4^	2.00 × 10^3^	2.58 × 10^5^	0.17
CECF20	2.30 × 10^3^	**2.19 × 10^3^**	2.11 × 10^3^	**48.30**	2.35 × 10^3^	2.20 × 10^3^	2.05 × 10^3^	76.80	0.69
CECF21	2.37 × 10^3^	**2.25 × 10^3^**	2.21 × 10^3^	**40.60**	2.38 × 10^3^	2.34 × 10^3^	2.22 × 10^3^	47.90	**8.19 × 10^−5^**
CECF22	2.62 × 10^3^	**2.42 × 10^3^**	2.31 × 10^3^	**94.40**	4.00 × 10^3^	2.43 × 10^3^	2.24 × 10^3^	4.16 × 10^2^	**1.11 × 10^−3^**
CECF23	2.70 × 10^3^	2.67 × 10^3^	2.64 × 10^3^	**16.40**	2.70 × 10^3^	2.65 × 10^3^	2.62 × 10^3^	19.50	7.71 × 10^−4^
CECF24	2.81 × 10^3^	**2.75 × 10^3^**	2.55 × 10^3^	78.20	2.85 × 10^3^	2.79 × 10^3^	2.76 × 10^3^	24.70	**5.32 × 10^−3^**
CECF25	3.28 × 10^3^	3.06 × 10^3^	2.95 × 10^3^	93.70	2.97 × 10^3^	2.94 × 10^3^	2.68 × 10^3^	52.80	4.73 × 10^−6^
CECF26	4.12 × 10^3^	**3.33 × 10^3^**	2.82 × 10^3^	**2.79 × 10^2^**	4.68 × 10^3^	3.62 × 10^3^	2.88 × 10^3^	5.74 × 10^2^	**1.48 × 10^−2^**
CECF27	3.24 × 10^3^	**3.14 × 10^3^**	3.11 × 10^3^	**33.60**	3.24 × 10^3^	3.15 × 10^3^	3.09 × 10^3^	43.40	0.72
CECF28	3.74 × 10^3^	3.49 × 10^3^	3.18 × 10^3^	**1.13 × 10^2^**	3.74 × 10^3^	3.48 × 10^3^	3.22 × 10^3^	1.66 × 10^2^	0.86
CECF29	3.60 × 10^3^	**3.36 × 10^3^**	3.25 × 10^3^	**76.00**	3.73 × 10^3^	3.41 × 10^3^	3.17 × 10^3^	1.18 × 10^2^	**0.13**
CECF30	3.88 × 10^6^	**6.53 × 10^5^**	5.35 × 10^4^	**9.29 × 10^5^**	6.04 × 10^6^	1.47 × 10^6^	5.82 × 10^3^	1.49 × 10^6^	**1.96 × 10^−2^**

**Table 7:** Experimental results of RWOA and WOA on CEC-2022.

Function	WOA		RWOA		
	Mean	SD	Mean	SD	*p*-Values
CF1	2.19 × 10^4^	9.96 × 10^3^	**2.40 × 10^3^**	**9.83 × 10^2^**	**1.73** × **10^−6^**
CF2	4.53 × 10^2^	58.50	5.52 × 10^2^	1.14 × 10^2^	1.15 × 10^−4^
CF3	6.40 × 10^2^	13.30	**6.35 × 10^2^**	**10.80**	**1.75 × 10^−2^**
CF4	8.41 × 10^2^	13.30	**8.34 × 10^2^**	**5.40**	**4.70 × 10^−3^**
CF5	1.47 × 10^3^	3.70 × 10^2^	**1.35 × 10^3^**	**1.80 × 10^2^**	**0.27**
CF6	4.67 × 10^3^	1.68 × 10^3^	2.14 × 10^4^	3.13 × 10^4^	1.60 × 10^−4^
CF7	2.07 × 10^3^	22.90	2.10 × 10^3^	26.90	7.16 × 10^−4^
CF8	2.23 × 10^3^	7.69	**2.23 × 10^3^**	**5.82**	**3.60** × **10^−3^**
CF9	2.61 × 10^3^	50.40	2.63 × 10^3^	**40.40**	0.15
CF10	2.63 × 10^3^	2.55 × 10^2^	**2.57 × 10^3^**	**79.60**	**0.47**
CF11	2.98 × 10^3^	91.30	**2.96 × 10^3^**	2.58 × 10^2^	0.57
CF12	2.90 × 10^3^	45.30	**2.90 × 10^3^**	**25.80**	0.85

**Table 8:** Performance comparisons among four algorithms.

	TSA [6]		ABC [6]		WOA		RWOA	
	Mean	SD	Mean	SD	Mean	SD	Mean	SD
TF1	**7.64 × 10^−243^**	**0.00**	2.25 × 10^−17^	8.00 × 10^−18^	7.33 × 10^−89^	2.86 × 10^−88^	2.14 × 10^−190^	0.00
TF2	**3.49 × 10^−147^**	**1.75 × 10^−146^**	7.76 × 10^−17^	2.06 × 10^−17^	3.65 × 10^−59^	1.30 × 10^−58^	6.86 × 10^−106^	3.53 × 10^−105^
TF3	9.47 × 10^−63^	5.15 × 10^−62^	2.08 × 10^−14^	4.56 × 10^−14^	9.70 × 10^−5^	3.28 × 10^−4^	**1.05 × 10^−78^**	**3.98 × 10^−78^**
TF4	3.33 × 10^−2^	0.18	**0.00**	**0.00**	7.64 × 10^−6^	1.00 × 10^−5^	1.34 × 10^−4^	2.02 × 10^−4^
TF5	5.16 × 10^−4^	3.05 × 10^−4^	2.39 × 10^−3^	1.22 × 10^−3^	1.20 × 10^−3^	1.30 × 10^−3^	**5.75 × 10^−5^**	**8.73 × 10^−5^**
TF6	0.32	1.05	**3.42 × 10^−2^**	**3.57 × 10^−2^**	1.88	3.63	1.21	0.50
TF7	0.00	0.00	0.00	0.00	0.00	0.00	0.00	0.00
TF8	2.02 × 10^−2^	1.57 × 10^−2^	1.23 × 10^−3^	2.80 × 10^−3^	4.65 × 10^−2^	9.47 × 10^−2^	**0.00**	**0.00**
TF9	**7.11 × 10^−16^**	1.45 × 10^−15^	3.20 × 10^−15^	1.08 × 10^−15^	3.49 × 10^−15^	1.85 × 10^−15^	8.88 × 10^−16^	**0.00**
TF10	**9.42 × 10^−32^**	**3.34 × 10^−47^**	0.21	8.82 × 10^−18^	3.11 × 10^−4^	0.00	1.85 × 10^−4^	1.92 × 10^−4^

**Table 9:** Algorithm parameter settings.

Algorithm	Parameters	Year	Refs.
PSO	$\omega =0.2\sim 0.9{,\;c}_{1}{=c}_{2\;}=2$	1995	[1]
CS	pa = 0.25, α=1, β=1.5	2009	[3]
KHA	$V_{f}=0.02{,\;D}_{\max}=0.005{,\;N}_{\max}=0.01$	2012	[4]
GWO	α=2→0, linearly decrease	2014	[5]
SCA	α=2	2016	[7]
WOA	b = 1, α=2→0, linearly decrease	2016	[8]
MPA	P = 0.5, FADs = 0.2	2020	[9]
DBO	α=1 or−1, b = 0.3, k = 0.1, S = 0.5, P_percent = 0.2	2022	[10]

**Table 10:** Comparison of RWOA with other algorithms on F1–F13 with 10 dimensions.

Function	Parameters	CS	DBO	GWO	KH	PSO	SCA	WOA	MPA	RWOA
F1	Mean	2.71 × 10^−3^	1.26 × 10^−104^	2.49 × 10^−64^	0.00	7.01 × 10^−23^	1.33 × 10^−12^	9.30 × 10^−85^	4.85 × 10^−30^	**2.36 × 10^−181^**
	SD	1.71 × 10^−3^	6.91 × 10^−104^	8.63 × 10^−64^	0.00	1.66 × 10^−22^	3.94 × 10^−12^	2.70 × 10^−84^	6.15 × 10^−30^	**0.00**
	*p*-values	1.73 × 10^−6^	1.73 × 10^−6^	1.73 × 10^−6^	1.73 × 10^−6^	1.73 × 10^−6^	1.73 × 10^−6^	1.73 × 10^−6^	1.73 × 10^−6^	NA
F2	Mean	2.28 × 10^−5^	1.11 × 10^−51^	9.00 × 10^−37^	2.81 × 10^−170^	6.34 × 10^−13^	1.17 × 10^−9^	1.88 × 10^−54^	3.29 × 10^−17^	**6.86 × 10^−105^**
	SD	3.44 × 10^−5^	6.05 × 10^−51^	2.11 × 10^−36^	0.00	8.23 × 10^−13^	3.29 × 10^−9^	6.81 × 10^−54^	3.85 × 10^−17^	**1.94 × 10^−104^**
	*p*-values	1.73 × 10^−6^	1.73 × 10^−6^	1.73 × 10^−6^	1.73 × 10^−6^	1.73 × 10^−6^	1.73 × 10^−6^	1.73 × 10^−6^	1.73 × 10^−6^	NA
F3	Mean	3.29 × 10^−3^	7.59 × 10^−81^	1.19 × 10^−27^	0.00	3.26 × 10^−7^	2.10 × 10^−3^	1.85 × 10^2^	3.03 × 10^−14^	**1.25 × 10^−138^**
	SD	3.15 × 10^−3^	4.16 × 10^−80^	6.32 × 10^−27^	0.00	6.30 × 10^−7^	8.88 × 10^−3^	4.17 × 10^2^	6.51 × 10^−14^	**6.86 × 10^−138^**
	*p*-values	1.73 × 10^−6^	2.88 × 10^−6^	1.73 × 10^−6^	1.73 × 10^−6^	1.73 × 10^−6^	1.73 × 10^−6^	1.73 × 10^−6^	1.73 × 10^−6^	NA
F4	Mean	2.09 × 10^−3^	9.85 × 10^−48^	9.58 × 10^−21^	6.79 × 10^−165^	2.52 × 10^−6^	4.59 × 10^−4^	0.68	1.16 × 10^−12^	**2.07 × 10^−77^**
	SD	1.73 × 10^−3^	5.39 × 10^−47^	1.38 × 10^−20^	0.00	3.19 × 10^−6^	8.99 × 10^−4^	1.62	1.02 × 10^−12^	**7.49 × 10^−77^**
	*p*-values	1.73 × 10^−6^	2.60 × 10^−6^	1.73 × 10^−6^	1.73 × 10^−6^	1.73 × 10^−6^	1.73 × 10^−6^	1.73 × 10^−6^	1.73 × 10^−6^	NA
F5	Mean	1.89 × 10^−4^	4.58	6.50	8.59	8.71	7.37	6.63	1.56	6.75
	SD	2.92 × 10^−4^	0.82	0.56	2.96 × 10^−2^	1.5.60	0.29	0.62	0.38	0.47
	*p*-values	1.73 × 10^−6^	1.92 × 10^−6^	7.19 × 10^−2^	1.73 × 10^−6^	0.15	4.07 × 10^−5^	0.60	1.73 × 10^−6^	NA
F6	Mean	2.68 × 10^−3^	3.36 × 10^−32^	3.05 × 10^−6^	0.96	5.70 × 10^−23^	0.41	3.64 × 10^−4^	1.13 × 10^−12^	6.05 × 10^−3^
	SD	2.15 × 10^−3^	6.84 × 10^−32^	1.12 × 10^−6^	0.29	1.29 × 10^−22^	0.11	2.96 × 10^−4^	8.15 × 10^−13^	4.23 × 10^−3^
	*p*-values	1.59 × 10^−3^	1.73 × 10^−6^	1.73 × 10^−6^	1.73 × 10^−6^	1.73 × 10^−6^	1.73 × 10^−6^	1.73 × 10^−6^	1.73 × 10^−6^	NA
F7	Mean	1.65 × 10^−7^	1.05 × 10^−3^	5.65 × 10^−4^	9.90 × 10^−5^	6.11 × 10^−3^	2.02 × 10^−3^	1.78 × 10^−3^	7.35 × 10^−4^	**7.35 × 10^−5^**
	SD	1.42 × 10^−7^	5.64 × 10^−4^	4.47 × 10^−4^	7.15 × 10^−5^	2.95 × 10^−3^	1.54 × 10^−3^	2.12 × 10^−3^	4.73 × 10^−4^	9.56 × 10^−5^
	*p*-values	1.73 × 10^−6^	1.73 × 10^−6^	3.52 × 10^−6^	8.59 × 10^−2^	1.73 × 10^−6^	1.73 × 10^−6^	1.73 × 10^−6^	2.13 × 10^−6^	NA
F8	Mean	0.61	−3.63 × 10^3^	−2.74 × 10^3^	−1.59 × 10^3^	−2.42 × 10^3^	−2.20 × 10^3^	−3.45 × 10^3^	−3.57 × 10^3^	**−4.07 × 10^3^**
	SD	0.52	4.47 × 10^2^	2.71 × 10^2^	3.19 × 10^2^	3.31 × 10^2^	1.62 × 10^2^	5.89 × 10^2^	2.16 × 10^2^	2.74 × 10^2^
	*p*-values	1.73 × 10^−6^	6.32 × 10^−5^	1.92 × 10^−6^	1.73 × 10^−6^	1.73 × 10^−6^	1.73 × 10^−6^	6.32 × 10^−5^	6.34 × 10^−6^	NA
F9	Mean	2.77 × 10^−6^	1.38	0.10	0.12	0.00	5.15	0.24	0.71	**0.00**
	SD	1.86 × 10^−6^	4.47	0.56	0.56	0.00	3.27	1.32	3.87	**0.00**
	*p*-values	1.73 × 10^−6^	0.13	0.25	1.00	1.73 × 10^−6^	2.69 × 10^−5^	1.00	0.50	NA
F10	Mean	1.50 × 10^−4^	8.88 × 10^−16^	6.81 × 10^−15^	8.88 × 10^−16^	6.02 × 10^−12^	2.43 × 10^−7^	5.03 × 10^−15^	5.27 × 10^−15^	**8.88 × 10^−16^**
	SD	9.47 × 10^−5^	0.00	1.70 × 10^−15^	0.00	7.74 × 10^−12^	5.67 × 10^−7^	2.30 × 10^−15^	1.53 × 10^−15^	**0.00**
	*p*-values	1.73 × 10^−6^	1.00	6.25 × 10^−7^	1.00	1.73 × 10^−6^	1.73 × 10^−6^	3.56 × 10^−6^	4.00 × 10^−7^	NA
F11	Mean	1.05	2.34 × 10^−2^	3.51 × 10^−2^	0.00	0.23	7.88 × 10^−2^	4.16 × 10^−2^	0.00	**0.00**
	SD	0.90	5.33 × 10^−2^	6.51 × 10^−2^	0.00	0.16	0.14	8.49 × 10^−2^	0.00	**0.00**
	*p*-values	1.73 × 10^−6^	1.56 × 10^−2^	8.86 × 10^−5^	1.00	1.73 × 10^−6^	1.73 × 10^−6^	7.81 × 10^−3^	1.00	NA
F12	Mean	4.51 × 10^−4^	5.19 × 10^−12^	3.92 × 10^−3^	0.36	1.33 × 10^−24^	8.97 × 10^−2^	5.03 × 10^−3^	5.06 × 10^−13^	2.55 × 10^−3^
	SD	3.15 × 10^−4^	2.83 × 10^−11^	7.98 × 10^−3^	0.22	3.77 × 10^−24^	3.05 × 10^−2^	8.99 × 10^−3^	3.86 × 10^−13^	2.11 × 10^−3^
	*p*-values	4.29 × 10^−6^	1.73 × 10^−6^	0.16	1.73 × 10^−6^	1.73 × 10^−6^	1.73 × 10^−6^	0.54	1.73 × 10^−6^	NA
F13	Mean	5.27 × 10^−4^	3.75 × 10^−4^	3.39 × 10^−3^	0.76	1.62 × 10^−22^	0.28	1.55 × 10^−2^	3.22 × 10^−12^	1.67 × 10^−2^
	SD	3.60 × 10^−4^	2.00 × 10^−3^	1.85 × 10^−2^	0.18	6.35 × 10^−22^	8.24 × 10^−2^	2.92 × 10^−2^	3.16 × 10^−12^	1.16 × 10^−2^
	*p*-values	1.73 × 10^−6^	1.73 × 10^−6^	3.11 × 10^−5^	1.73 × 10^−6^	1.73 × 10^−6^	1.73 × 10^−6^	5.71 × 10^−2^	1.73 × 10^−6^	NA

Note: NA in the table indicates invalid data.

**Table 11:** Comparison of RWOA with other algorithms on F1–F13 with 50 dimensions.

Function	Parameters	CS	DBO	GWO	KH	PSO	SCA	WOA	MPA	RWOA
F1	Mean	1.86 × 10^2^	1.72 × 10^−108^	3.46 × 10^−22^	0.00	9.18 × 10^−2^	5.71 × 10^2^	2.89 × 10^−79^	1.69 × 10^−20^	2.04 × 10^−180^
	SD	9.51 × 10^2^	9.44 × 10^−108^	2.68 × 10^−22^	0.00	8.33 × 10^−2^	7.49 × 10^2^	1.13 × 10^−78^	1.51 × 10^−20^	0.00
	*p*-values	1.73 × 10^−6^	1.73 × 10^−6^	1.73 × 10^−6^	1.73 × 10^−6^	1.73 × 10^−6^	1.73 × 10^−6^	1.73 × 10^−6^	1.73 × 10^−6^	NA
F2	Mean	0.10	2.49 × 10^−57^	1.21 × 10^−13^	4.63 × 10^−171^	0.89	0.56	2.31 × 10^−52^	4.69 × 10^−12^	4.65 × 10^−102^
	SD	0.11	1.25 × 10^−56^	6.37 × 10^−14^	0.00	0.49	0.58	6.12 × 10^−52^	4.11 × 10^−12^	1.24 × 10^−101^
	*p*-values	1.73 × 10^−6^	1.73 × 10^−6^	1.73 × 10^−6^	1.73 × 10^−6^	1.73 × 10^−6^	1.73 × 10^−6^	1.73 × 10^−6^	1.73 × 10^−6^	NA
F3	Mean	15.60	8.44 × 10^−13^	3.84 × 10^−2^	0.00	1.21 × 10^3^	4.38 × 10^4^	1.77 × 10^5^	0.13	1.55 × 10^−121^
	SD	17.80	4.62 × 10^−12^	6.26 × 10^−2^	0.00	3.25 × 10^2^	1.36 × 10^4^	3.56 × 10^4^	0.28	8.46 × 10^−121^
	*p*-values	1.73 × 10^−6^	4.29 × 10^−6^	1.73 × 10^−6^	1.73 × 10^−6^	1.73 × 10^−6^	1.73 × 10^−6^	1.73 × 10^−6^	1.73 × 10^−6^	NA
F4	Mean	7.86 × 10^2^	1.11 × 10^−50^	9.06 × 10^−5^	3.02 × 10^−167^	3.27	68.10	59.90	3.53 × 10^−8^	9.18 × 10^−75^
	SD	4.24 × 10^3^	6.08 × 10^−50^	6.86 × 10^−5^	0.00	0.52	7.22	28.50	1.37 × 10^−8^	3.46 × 10^−74^
	*p*-values	1.73 × 10^−6^	1.73 × 10^−6^	1.73 × 10^−6^	1.73 × 10^−6^	1.73 × 10^−6^	1.73 × 10^−6^	1.73 × 10^−6^	1.73 × 10^−6^	NA
F5	Mean	0.98	45.70	47.30	48.50	3.27 × 10^2^	3.50 × 10^6^	48.00	46.20	47.70
	SD	1.42	0.25	0.82	1.51 × 10^−2^	2.10 × 10^2^	4.08 × 10^6^	0.39	0.51	0.30
	*p*-values	1.73 × 10^−6^	1.73 × 10^−6^	6.87 × 10^−2^	1.92 × 10^−6^	1.73 × 10^−6^	1.73 × 10^−6^	1.48 × 10^−4^	1.92 × 10^−6^	NA
F6	Mean	9.43	1.58 × 10^−2^	2.25	10.60	7.24 × 10^−2^	4.34 × 10^2^	0.69	0.27	0.47
	SD	16.10	3.69 × 10^−2^	0.56	0.68	3.85 × 10^−2^	4.25 × 10^2^	0.21	0.16	0.25
	*p*-values	5.79 × 10^−5^	1.73 × 10^−6^	1.73 × 10^−6^	1.73 × 10^−6^	1.92 × 10^−6^	1.73 × 10^−6^	2.96 × 10^−3^	6.64 × 10^−4^	NA
F7	Mean	1.46 × 10^−3^	2.09 × 10^−3^	2.49 × 10^−3^	1.11 × 10^−4^	1.85	3.47	2.66 × 10^−3^	9.50 × 10^−4^	**4.27 × 10** **^−5^**
	SD	1.29 × 10^−3^	1.25 × 10^−3^	9.91 × 10^−4^	9.99 × 10^−5^	0.78	4.23	2.84 × 10^−3^	5.65 × 10^−4^	3.63 × 10^−5^
	*p*-values	2.88 × 10^−6^	1.73 × 10^−6^	1.73 × 10^−6^	8.31 × 10^−4^	1.73 × 10^−6^	1.73 × 10^−6^	1.73 × 10^−6^	1.73 × 10^−6^	NA
F8	Mean	7.19 × 10^6^	−1.41 × 10^4^	−9.14 × 10^3^	−3.34 × 10^3^	−7.53 × 10^3^	−4.87 × 10^3^	−1.86 × 10^4^	−1.33 × 10^4^	**−2.06 × 10^4^**
	SD	1.89 × 10^7^	2.64 × 10^3^	1.26 × 10^3^	6.65 × 10^2^	2.31 × 10^3^	3.71 × 10^2^	2.72 × 10^3^	7.24 × 10^2^	8.46 × 10^2^
	*p*-values	1.73 × 10^−6^	1.73 × 10^−6^	1.73 × 10^−6^	1.73 × 10^−6^	1.73 × 10^−6^	1.73 × 10^−6^	4.20 × 10^−4^	1.73 × 10^−6^	NA
F9	Mean	2.68 × 10^−2^	1.26	2.49	0.00	1.40 × 10^2^	1.08 × 10^2^	0.00	0.00	**0.00**
	SD	2.37 × 10^−2^	4.82	2.95	0.00	33.50	63.00	0.00	0.00	0.00
	*p*-values	1.73 × 10^−6^	0.50	1.73 × 10^−6^	1.00	1.73 × 10^−6^	1.73 × 10^−6^	1.00	1.00	NA
F10	Mean	1.17	8.88 × 10^−16^	3.19 × 10^−12^	8.88 × 10^−16^	1.44	1.89 × 10^1^	4.20 × 10^−15^	1.89 × 10^−11^	**8.88 × 10** **^−16^**
	SD	1.39	0.00	1.73 × 10^−12^	0.00	0.57	4.54	2.27 × 10^−15^	1.05 × 10^−11^	0.00
	*p*-values	1.73 × 10^−6^	1.00	1.73 × 10^−6^	1.00	1.73 × 10^−6^	1.73 × 10^−6^	8.19 × 10^−6^	1.73 × 10^−6^	NA
F11	Mean	5.10 × 10^7^	0.00	3.31 × 10^−3^	0.00	6.86 × 10^−3^	4.93	0.00	0.00	**0.00**
	SD	1.27 × 10^8^	0.00	7.15 × 10^−3^	0.00	7.87 × 10^−3^	2.68	0.00	0.00	0.00
	*p*-values	1.73 × 10^−6^	1.00	7.81 × 10^−3^	1.00	1.73 × 10^−6^	1.73 × 10^−6^	1.00	1.00	NA
F12	Mean	2.02	2.30 × 10^−3^	0.11	1.01	2.26 × 10^−2^	1.44 × 10^7^	1.74 × 10^−2^	6.73 × 10^−3^	1.07 × 10^−2^
	SD	2.02	1.14 × 10^−2^	9.51 × 10^−2^	0.13	5.52 × 10^−2^	2.43 × 10^7^	1.03 × 10^−2^	5.39 × 10^−3^	5.96 × 10^−3^
	*p*-values	1.73 × 10^−6^	3.11 × 10^−5^	1.73 × 10^−6^	1.73 × 10^−6^	7.19 × 10^−2^	1.73 × 10^−6^	9.27 × 10^−3^	1.29 × 10^−3^	NA
F13	Mean	2.21	1.34	1.84	4.94	8.34 × 10^−2^	1.34 × 10^7^	0.83	0.38	0.25
	SD	1.93	0.73	0.31	1.57 × 10^−4^	6.15 × 10^−2^	1.92 × 10^7^	0.31	0.20	0.10
	*p*-values	3.88 × 10^−6^	2.13 × 10^−6^	1.73 × 10^−6^	1.73 × 10^−6^	7.69 × 10^−6^	1.73 × 10^−6^	1.73 × 10^−6^	8.94 × 10^−4^	NA

**Table 12:** Comparison of RWOA with other algorithms on F1–F13 with 100 dimensions.

Function	Parameters	CS	DBO	GWO	KH	PSO	SCA	WOA	MPA	RWOA
F1	Mean	7.25 × 10^3^	1.56 × 10^−122^	4.36 × 10^−14^	0.00	16.60	9.84 × 10^3^	1.90 × 10^−78^	7.29 × 10^−19^	1.67 × 10^−179^
	SD	3.77 × 10^4^	7.15 × 10^−122^	2.72 × 10^−14^	0.00	6.22	5.70 × 10^3^	1.01 × 10^−77^	5.87 × 10^−19^	0.00
	*p*-values	1.73 × 10^−6^	1.73 × 10^−6^	1.73 × 10^−6^	1.73 × 10^−6^	1.73 × 10^−6^	1.73 × 10^−6^	1.73 × 10^−6^	1.73 × 10^−6^	NA
F2	Mean	0.33	8.00 × 10^−54^	6.36 × 10^−9^	2.21 × 10^−169^	28.00	8.27	3.86 × 10^−52^	4.41 × 10^−11^	1.16 × 10^−99^
	SD	0.38	4.38 × 10^−53^	1.82 × 10^−9^	0.00	6.53	5.84	8.77 × 10^−52^	4.39 × 10^−11^	5.14 × 10^−99^
	*p*-values	1.73 × 10^−6^	1.73 × 10^−6^	1.73 × 10^−6^	1.73 × 10^−6^	1.73 × 10^−6^	1.73 × 10^−6^	1.73 × 10^−6^	1.73 × 10^−6^	NA
F3	Mean	19.70	3.64 × 10^−32^	2.81 × 10^2^	0.00	1.44 × 10^4^	2.35 × 10^5^	9.50 × 10^5^	10.80	7.60 × 10^−107^
	SD	26.00	1.99 × 10^−31^	2.57 × 10^2^	0.00	2.95 × 10^3^	6.43 × 10^4^	2.05 × 10^5^	10.70	4.15 × 10^−106^
	*p*-values	1.73 × 10^−6^	8.47 × 10^−6^	1.73 × 10^−6^	1.73 × 10^−6^	1.73 × 10^−6^	1.73 × 10^−6^	1.73 × 10^−6^	1.73 × 10^−6^	NA
F4	Mean	37.00	9.71 × 10^−55^	0.36	8.12 × 10^−167^	10.80	88.70	73.60	3.22 × 10^−7^	2.33 × 10^−74^
	SD	1.34 × 10^2^	5.18 × 10^−54^	0.36	0.00	1.67	2.73	24.30	1.17 × 10^−7^	1.18 × 10^−73^
	*p*-values	1.73 × 10^−6^	1.73 × 10^−6^	1.73 × 10^−6^	1.73 × 10^−6^	1.73 × 10^−6^	1.73 × 10^−6^	1.73 × 10^−6^	1.73 × 10^−6^	NA
F5	Mean	2.90	96.90	97.60	98.20	1.04 × 10^4^	9.68 × 10^7^	97.90	97.20	97.80
	SD	3.12	0.82	0.79	1.36 × 10^−2^	3.49 × 10^3^	4.82 × 10^7^	0.31	0.75	0.32
	*p*-values	1.73 × 10^−6^	1.06 × 10^−4^	0.49	1.73 × 10^−6^	1.73 × 10^−6^	1.73 × 10^−6^	5.45 × 10^−2^	1.48 × 10^−3^	NA
F6	Mean	1.79 × 10^3^	2.60	9.30	23.10	13.30	9.67 × 10^3^	2.37	4.80	**1.25**
	SD	9.72 × 10^3^	0.52	0.76	0.63	4.36	5.75 × 10^3^	0.85	0.74	0.54
	*p*-values	4.07 × 10^−5^	3.18 × 10^−6^	1.73 × 10^−6^	1.73 × 10^−6^	1.73 × 10^−6^	1.73 × 10^−6^	6.32 × 10^−5^	1.73 × 10^−6^	NA
F7	Mean	3.83 × 10^−3^	1.68 × 10^−3^	5.60 × 10^−3^	1.06 × 10^−4^	1.48 × 10^3^	1.33 × 10^2^	1.99 × 10^−3^	1.67 × 10^−3^	**4.68 × 10** **^−^** **^5^**
	SD	4.59 × 10^−3^	1.65 × 10^−3^	2.22 × 10^−3^	9.41 × 10^−5^	2.27 × 10^2^	84.10	2.34 × 10^−3^	7.41 × 10^−4^	6.72 × 10^−5^
	*p*-values	3.52 × 10^−6^	1.73 × 10^−6^	1.73 × 10^−6^	1.11 × 10^−3^	1.73 × 10^−6^	1.73 × 10^−6^	1.92 × 10^−6^	1.73 × 10^−6^	NA
F8	Mean	1.10 × 10^7^	−2.77 × 10^4^	−1.71 × 10^4^	−4.53 × 10^3^	−1.15 × 10^4^	−7.06 × 10^3^	−3.69 × 10^4^	−2.38 × 10^4^	** −4.16 ** **× 10^4^**
	SD	3.82 × 10^7^	6.83 × 10^3^	1.54 × 10^3^	6.40 × 10^2^	4.25 × 10^3^	5.02 × 10^2^	4.75 × 10^3^	1.27 × 10^3^	4.26 × 10^2^
	*p*-values	1.73 × 10^−6^	1.73 × 10^−6^	1.73 × 10^−6^	1.73 × 10^−6^	1.73 × 10^−6^	1.73 × 10^−6^	1.36 × 10^−5^	1.73 × 10^−6^	NA
F9	Mean	9.36 × 10^−2^	0.00	7.22	0.00	5.38 × 10^2^	2.62 × 10^2^	0.00	0.00	**0.00**
	SD	0.10	0.00	5.53	0.00	67.00	1.23 × 10^2^	0.00	0.00	0.00
	*p*-values	1.73 × 10^−6^	1.00	1.73 × 10^−6^	1.00	1.73 × 10^−6^	1.73 × 10^−6^	1.00	1.00	NA
F10	Mean	4.26	8.88 × 10^−16^	2.44 × 10^−8^	8.88 × 10^−16^	3.44	18.10	4.44 × 10^−15^	9.23 × 10^−11^	**8.88 × 10** **^−^** **^16^**
	SD	4.87	0.00	8.64 × 10^−9^	0.00	0.23	4.81	2.47 × 10^−15^	4.32 × 10^−11^	0.00
	*p*-values	1.73 × 10^−6^	1.00	1.73 × 10^−6^	1.00	1.73 × 10^−6^	1.73 × 10^−6^	1.14 × 10^−5^	1.73 × 10^−6^	
F11	Mean	4.57 × 10^8^	0.00	3.63 × 10^−3^	0.00	0.29	73.30	0.00	0.00	**0.00**
	SD	1.82 × 10^9^	0.00	8.27 × 10^−3^	0.00	6.33 × 10^−2^	52.90	0.00	0.00	0.00
	*p*-values	1.73 × 10^−6^	1.00	1.73 × 10^−6^	1.00	1.73 × 10^−6^	1.73 × 10^−6^	1.00	1.00	NA
F12	Mean	7.58	2.50 × 10^−2^	0.25	1.11	3.39	2.98 × 10^8^	2.82 × 10^−2^	6.08 × 10^−2^	**1.50 × 10** **^−^** **^2^**
	SD	9.82	9.14 × 10^−3^	7.61 × 10^−2^	9.29 × 10^−2^	1.64	1.23 × 10^8^	1.58 × 10^−2^	1.39 × 10^−2^	7.37 × 10^−3^
	*p*-values	2.13 × 10^−6^	3.88 × 10^−4^	1.73 × 10^−6^	1.73 × 10^−6^	1.73 × 10^−6^	1.73 × 10^−6^	8.92 × 10^−5^	1.73 × 10^−6^	NA
F13	Mean	5.01	7.50	6.39	9.91	49.10	4.52 × 10^8^	1.94	6.78	**0.72**
	SD	4.99	1.42	0.48	1.18 × 10^−3^	23.40	2.24 × 10^8^	0.80	2.49	0.27
	*p*-values	4.07 × 10^−5^	1.73 × 10^−6^	1.73 × 10^−6^	1.73 × 10^−6^	1.73 × 10^−6^	1.73 × 10^−6^	2.88 × 10^−6^	1.73 × 10^−6^	NA

## Data Availability

This manuscript does not report data generation or analysis.
